# Histotype-specific incidence and survival of urothelial carcinoma—an analysis of the German North Rhine-Westphalia Cancer Registry

**DOI:** 10.1016/j.esmorw.2025.100678

**Published:** 2026-01-19

**Authors:** C. Darr, L. Möller, A. Szentkiralyi, K. Claassen, N. Schürger, H. Reis, T. Hilser, A. Stang, B.A. Hadaschik, H. Kajüter, V. Grünwald

**Affiliations:** 1 Department of Urology, University Hospital Essen, Essen, Germany; 2 Cancer Registry of North Rhine-Westphalia, Bochum, Germany; 3 Dr. Senckenberg Institute of Pathology, University Hospital Frankfurt, Goethe University Frankfurt, Frankfurt, Germany; 4 Department of Medical Oncology, West German Cancer Center, University Hospital Essen, Essen, Germany; 5 Institute of Medical Informatics, Biometry, and Epidemiology, University Hospital Essen, Essen, Germany

**Keywords:** urothelial carcinoma, relative survival, registries, variant histology, Germany, rare cancer

## Abstract

**Background:**

The aim of this study was to evaluate the incidence and survival of urothelial carcinoma (UC) and non-urothelial tumor types. The primary objective was to define the incidence and survival of pure UC and non-UC tumor types.

**Materials and methods:**

Malignant invasive urinary cancers of the urothelial tract diagnosed between 2008 and 2022 were identified via the North Rhine-Westphalia Cancer Registry and classified according to the 2016 World Health Organization (WHO) classification (fourth edition). Evaluation focused on pure tumor types: UC, papillary invasive UC (PIUC), squamous-cell carcinoma (SCC), adenocarcinoma (ADC), rarer tumor types and mixed histologies grouped under other specific tumor types (OSTT), and unspecified tumor types (UTT). The primary outcomes were age-standardized incidence rate with estimated annual percentage changes, and relative survival.

**Results:**

UC and PIUC were the most common histology types, accounting for 57.9% and 26.2% (*n* = 73 751), respectively. The age-standardized incidence of PIUC and UTT decreased over time in both sexes, while OSTT incidence increased over time. Incidence rate declined among men with SCC and among women with ADC. Relative survival was notably poor for SCC (32.3%) and OSTT (23.8%), in contrast to a more favorable outcome for PIUC (72.0%). Decreasing survival with advancing T-stages was noted, particularly for UC, PIUC, ADC, and UTT; T1 in PIUC showed the best survival (81.0%) and T3-4 in OSTT had the poorest survival (16.4%).

**Conclusion:**

Relative survival was most favorable for PIUC, UC, and ADC. In contrast, SSC and OSTT exhibited poorer survival outcomes, highlighting a pressing medical need for enhanced treatment options for these subgroups.

## Highlights

• Relative survival highly varied by histology.
• Worst survival was observed in OSTT.
• Best survival was observed in PIUC.
• Tumor stage strongly influenced survival, dropping from 76.1% (T1) to 33.7% (T3-4).

## Introduction

Urothelial carcinoma (UC) is a significant global health concern, ranking as the ninth most common cancer worldwide.^1^ It exhibits varying incidence and mortality rates across different regions, influenced by factors such as risk exposure, detection practices, and treatment availability. For instance, in Western Europe, the age-standardized incidence rate (ASR; world standard population) is higher for men (19.8 per 100 000 person-years) compared with women (5.4 per 100 000 person-years). Worldwide, the age-standardized mortality rate (world standard population) for bladder cancer is 3.1 for men and 0.8 for women per 100 000 person-years.^1^^,^^2^

A review of cystectomy specimens by a uropathologist revealed that ∼67% of bladder cancer cases were classified as pure UC, and the remaining 33% consisted of histologic UC variants.^3^ The classification of UC into different histopathological variants, as per the 2022 World Health Organization (WHO) classification, is crucial for understanding its biological behavior and clinical implications. Variants of UC such as squamous differentiation and adenocarcinoma (ADC) are distinct from pure UC, with varying prognoses and treatment requirements. Morphologic papillary invasive urothelial carcinoma (PIUC) has not yet been studied separately, as this morphology subclass has been merged with UC in previous literature.

Accurate diagnosis of these subtypes is essential for tailored treatment strategies and prognostication, emphasizing the need for precise histopathological assessment.^3^, ^4^, ^5^ Despite the importance of UC subtypes, current research predominantly focuses on pure UC, often excluding variant histologies in clinical trials.^6^ This exclusion results in significant data gaps, particularly for rare subtypes. Recent recommendations from the Society for Immunotherapy of Cancer and the International Bladder Cancer Group advocate for the inclusion of histologic variants in trial designs, yet few studies have addressed this need.^7^^,^^8^ This oversight underscores the urgency for comprehensive research that encompasses all UC subtypes to ensure equitable advancements in diagnosis and treatment.

This study aims to define the incidence and survival of pure UC and non-UC tumor types, thereby supporting the development of modern therapeutic approaches and providing a foundation for future prospective clinical trials in these rare patient cohorts.

## Materials and methods

North Rhine-Westphalia (NRW) is the most populated federal state in Germany (18.1 million inhabitants). Cancer reporting to the Cancer Registry of NRW has been mandatory since 2005. The database of the state cancer registry in NRW includes comprehensive information on patient demographics, tumor diagnosis, tumor characteristics, and mortality follow-up. In addition, this also includes the International Classification of Diseases for Oncology (ICD-O) codes with corresponding morphological data from the pathology reports.

### Design and selection criteria

Patients diagnosed in NRW with malignant invasive urinary cancers including cancers of the renal pelvis [International Classification of Diseases (ICD)-10: C65], ureter (C66), urinary bladder (C67), and other or unspecified urinary organs (C68) from 2008 to 2022 were selected. WHO classification and allocation of the corresponding coding was adjusted twice during the observation period. This was taken into account when processing the datasets by grouping them into the morphologies to be examined. Patients were divided into six groups: invasive UC, PIUC, pure squamous-cell carcinoma (SCC), pure ADC, other specific tumor types (OSTT) with a frequency of <1%, and unspecified tumor types (UTT) according to the 2016 WHO classification (fourth edition).^9^ Classification was based on the ICD-O-3 codes provided by the pathology report. Pure cancers were included according to the morphology classification. Mixed morphologies were grouped under OSTT to account for histological heterogeneity. Non-invasive tumors (ICD-10: D41.1-D41.4, D09.0, D09.1, *n* = 57 757) as well as very rare variants (*n* < 5) were excluded. Tumors with ICD-O-3 code 8130/3 and 8131/3 (PIUC) were analyzed separately because of their favorable prognosis. T-stages were classified according to the criteria of the Union for International Cancer Control.^10^ Comprehensive mortality follow-up for cancer patients was routinely assessed through validated record linkage with electronic reports on all deceased individuals in NRW obtained from the population registry.

### Statistical analysis

ASRs were calculated with all cases diagnosed in the respective period as numerator and the sum of the annual mid-year population as denominator. Age standardization was carried out using the old European Standard.^11^ For detailed analysis of incidence time trends, estimated annual percentage changes (APCs) were determined for the histological types and in relation to sex. The APC was calculated by fitting a regression line to the natural logarithm of the annual ASR using the calendar years as predictor variables: Y = a + bx, where Y = ln(ASR), x is the calendar year, and APC = 100 × (e^b^ − 1).

For survival analysis we calculated 5-year absolute and relative survival (RS). The number of patients in the survival analysis was reduced because 4941 (6.8%) cases were death certificate only, and the analysis was restricted to the calendar period 2018-2022. RS for a calendar period is defined as the ratio of the observed survival time of patients with urinary tract neoplasms (absolute survival) to the expected survival time of the general population of the same age, sex, and calendar period in NRW.^12^ This can be interpreted as the expected survival of patients with cancer under the hypothetical assumption that cancer is the only cause of death.^13^ Survival time per patient was the time interval between the date of diagnosis and death or end of the follow-up on 31 December 2022. Expected survival was estimated using the Ederer-II method based on life tables of NRW.^13^^,^^14^

RS was calculated using the period approach, since it provides more up-to-date survival estimates than the traditional cohort approach and therefore enables timely detection of changes in survival.^15^ We also estimated age-specific RS. All analyses were carried out with R, version 4.4.2, using the package ‘periodR’ for RS analysis.

## Results

Based on the inclusion criteria, 74 045 patients were identified. Of these, 294 were excluded due to uncertain biological behavior. The basic characteristics of the remaining 73 751 patients are presented in Table 1.

**Table 1:** Demographics and baseline characteristics of registered patients with malignant invasive urinary cancers of the years 2008-2022 in North Rhine-Westphalia, Germany

	UC *n* = 42 684 (57.9%) *n* (%)	PIUC *n* = 20 070 (27.2%) *n* (%)	SCC *n* = 1530 (2.1%) *n* (%)	ADC *n* = 928 (1.3%) n (%)	OSTT *n* = 1037 (1.4%) *n* (%)	UTT *n* = 7502 (10.2%) *n* (%)	Overall *n* = 73 751 (100.0%) *n* (%)
Sex							
Female	11 808 (27.7)	4337 (21.6)	942 (61.6)	386 (41.6)	342 (33.0)	2892 (38.5)	20 707 (28.1)
Male	30 876 (72.3)	15 733 (78.4)	588 (38.4)	542 (58.4)	695 (67.0)	4610 (61.5)	53 044 (71.9)
Age at diagnosis (years)							
Mean (SD)	73.0 (10.9)	72.7 (10.6)	72.2 (12.8)	68.9 (13.2)	72.4 (11.8)	77.9 (11.7)	73.4 (11.1)
Median (min-max)	74.0 (21.0-104)	74.0 (20.0-103)	74.0 (25.0-106)	70.0 (23.0-97.0)	74.0 (15.0-98.0)	80.0 (15.0-106)	75.0 (15.0-106)
Age group (years)							
15-59	5242 (12.3)	2404 (12.0)	269 (17.6)	208 (22.4)	145 (14.0)	594 (7.9)	8862 (12.0)
60-69	9211 (21.6)	4632 (23.1)	300 (19.6)	246 (26.5)	225 (21.7)	1015 (13.5)	15 629 (21.2)
70-79	14 955 (35.0)	7245 (36.1)	432 (28.2)	255 (27.5)	349 (33.7)	2004 (26.7)	25 240 (34.2)
≥80	13 276 (31.1)	5789 (28.8)	529 (34.6)	219 (23.6)	318 (30.7)	3889 (51.8)	24 020 (32.6)
ICD-10							
C65: Renal pelvic	2880 (6.7)	1360 (6.8)	75 (4.9)	32 (3.4)	23 (2.2)	262 (3.5)	4632 (6.3)
C66: Ureter	1572 (3.7)	746 (3.7)	29 (1.9)	31 (3.3)	32 (3.1)	133 (1.8)	2543 (3.4)
C67: Urinary bladder	36 984 (86.6)	17 772 (88.6)	1294 (84.6)	807 (87.0)	943 (90.9)	5959 (79.4)	63 759 (86.5)
C68: Other and unspecified urinary organs	1248 (2.9)	192 (1.0)	132 (8.6)	58 (6.3)	39 (3.8)	1148 (15.3)	2817 (3.8)
T-stage							
T1	11 959 (28.0)	12 744 (63.5)	236 (15.4)	189 (20.4)	71 (6.8)	364 (4.9)	25 563 (34.7)
T2	15 494 (36.3)	3708 (18.5)	517 (33.8)	197 (21.2)	331 (31.9)	329 (4.4)	20 576 (27.9)
T3	7166 (16.8)	1523 (7.6)	332 (21.7)	145 (15.6)	202 (19.5)	134 (1.8)	9502 (12.9)
T4	3057 (7.2)	379 (1.9)	183 (12.0)	84 (9.1)	88 (8.5)	98 (1.3)	3889 (5.3)
Unknown	5008 (11.7)	1716 (8.6)	262 (17.1)	313 (33.7)	345 (33.3)	6577 (87.7)	14 221 (19.3)

ADC, adenocarcinoma; ICD, International Classification of Diseases; OSTT, other specific tumor types; PIUC, papillary invasive urothelial carcinoma; SCC, squamous-cell carcinoma; SD, standard deviation; UC, urothelial carcinoma; UTT, unspecified tumor types.

**Figure 1:**
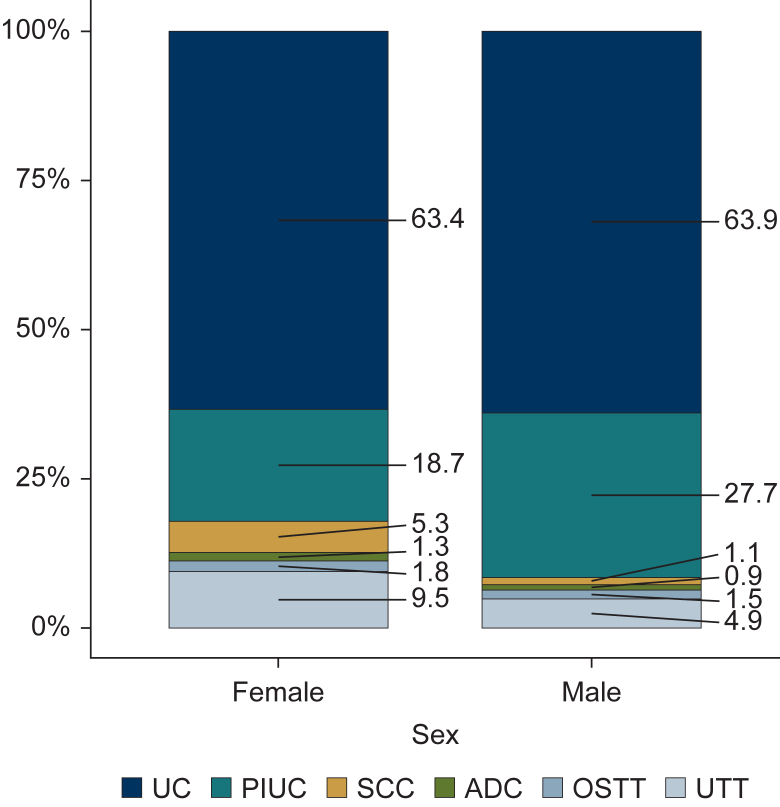
**Relative frequency of histology groups (%) of malignant invasive urinary cancers between 2020 and 2022, North Rhine-Westphalia, Germany.** ADC, adenocarcinoma; OSTT, other specific tumor types; PIUC, papillary urothelial carcinoma; SCC, squamous-cell carcinoma; UC, urothelial carcinoma; UTT, unspecified tumor types.

Overall, the distribution across subgroups showed that the majority of patients had UC (42 684, 57.9%), followed by PIUC (20 070, 27.2%), SCC (1530, 2.1%), ADC (928, 1.3%), OSTT (1037, 1.4%), and UTT (7502, 10.2%). In terms of demographic characteristics, no relevant differences were observed between the subgroups, and the distribution of tumor topography was comparable. The T-stage distribution varied across subgroups, with a high proportion of T1 in PIUC (63.5%), while T-stage was unknown in 87.5% of UTT. Women had a lower proportion of PIUC (18.7% versus 27.7%) and a higher proportion of SCC (5.3% versus 1.1%) and UTT (9.5% versus 4.9%) compared with men. The other groups were almost equally distributed across both sexes (Figure 1).

**Figure 2:**
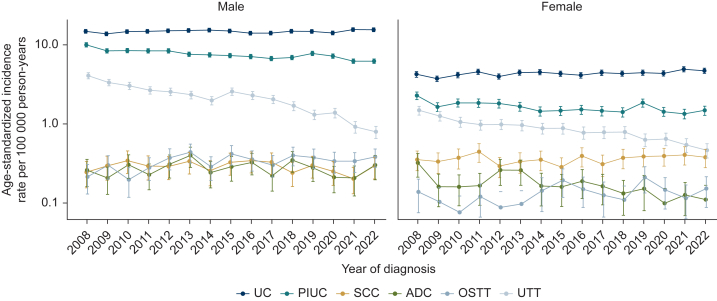
**Annual age-standardized incidence rate of urinary tract carcinoma for each histology group in North Rhine-Westphalia, Germany, between 2008 and 2022.^a^** ADC, adenocarcinoma; OSTT, other specific tumor types; PIUC, papillary invasive urothelial carcinoma; SCC, squamous-cell carcinoma; UC, urothelial carcinoma; UTT, unspecified tumor types. ^a^Using the old European Standard population.

ASR per 100 000 person-years was higher in men than in women over the years and showed a slight decrease over time especially among men (Supplementary Figure S1, available at https://doi.org/10.1016/j.esmorw.2025.100678). Based on the APC values (Table 2), the incidence increased for UC among women {0.9 [95% confidence interval (CI) 0.3-1.6]} and for OSTT among men [3.0 (95% CI 0.7-5.3)]. Decreasing incidence trends were shown for PIUC [women –2.5 (95% CI –3.2 to –1.8), men –2.5 (95% CI –3.2 to –1.8)], ADC [women –5.0 (95% CI –7.5 to –2.5)], and UTT [women –6.4 (95% CI –7.3 to –5.6), men –9.3 (95% CI –10.9 to –7.6)], while the incidence rates remained stable over time in the other subgroups (Table 2, Figure 2).

**Table 2:** ASRs^a^ and CRs per 100 000 person-years during the first and last 3-year period, and APC during the whole time period of registered patients with malignant invasive urinary cancers in North Rhine-Westphalia, Germany

		2008-2010	2020-2022	2008-2010	2020-2022	2008-2022
Histology	Sex	ASR (95% CI)	ASR (95% CI)	CR (95% CI)	CR (95% CI)	APC (95% CI)
UC	Male	14.4 (14.0 to 14.8)	15.1 (14.7 to 15.5)	21.2 (20.6 to 21.8)	25.7 (25.1 to 26.3)	0.2 (–0.2 to 0.7)
	Female	4.1 (3.9 to 4.2)	4.7 (4.5 to 4.9)	7.7 (7.3 to 8.0)	9.7 (9.3 to 10.1)	0.9 (0.3 to 1.6)
PIUC	Male	9.0 (8.6 to 9.3)	6.5 (6.3 to 6.8)	13.1 (12.6 to 13.5)	11.1 (10.7 to 11.5)	–2.5 (–3.2 to –1.8)
	Female	1.9 (1.8 to 2.1)	1.4 (1.3 to 1.5)	3.6 (3.4 to 3.9)	2.9 (2.7 to 3.1)	–2.3 (–3.5 to –1.1)
SCC	Male	0.3 (0.2 to 0.4)	0.2 (0.2 to 0.3)	0.4 (0.3 to 0.5)	0.4 (0.4 to 0.5)	–1.0 (–2.7 to 0.8)
	Female	0.4 (0.3 to 0.4)	0.4 (0.3 to 0.5)	0.6 (0.5 to 0.7)	0.8 (0.7 to 0.9)	0.7 (–0.7 to 2.2)
ADC	Male	0.3 (0.2 to 0.3)	0.2 (0.2 to 0.3)	0.4 (0.3 to 0.4)	0.4 (0.3 to 0.4)	–0.2 (–2.5 to 2.3)
	Female	0.2 (0.2 to 0.3)	0.1 (0.1 to 0.1)	0.3 (0.3 to 0.4)	0.2 (0.1 to 0.3)	–5.0 (–7.5 to –2.5)
OSTT	Male	0.2 (0.2 to 0.3)	0.4 (0.3 to 0.4)	0.3 (0.3 to 0.4)	0.6 (0.5 to 0.7)	3.0 (0.7 to 5.3)
	Female	0.1 (0.1 to 0.1)	0.1 (0.1 to 0.2)	0.2 (0.1 to 0.2)	0.3 (0.2 to 0.3)	2.9 (–0.1 to 6.0)
UTT	Male	3.5 (3.3 to 3.7)	1.0 (0.9 to 1.1)	5.1 (4.8 to 5.3)	2.0 (1.8 to 2.2)	–9.3 (–10.9 to –7.6)
	Female	1.3 (1.2 to 1.4)	0.6 (0.5 to 0.6)	2.9 (2.7 to 3.1)	1.4 (1.3 to 1.6)	–6.4 (–7.3 to –5.6)

ADC, adenocarcinoma; APC, annual percentage change; ASR, age-standardized incidence rate; CI, confidence interval; CR, crude incidence rate; OSTT, other specific tumor types; PIUC, papillary invasive urothelial carcinoma; SCC, squamous-cell carcinoma; UC, urothelial carcinoma; UTT, unspecified tumor types.

a Using the old European Standard population.

The 5-year RS within the 2018-2022 calendar period (*N* = 37 485) depended on histologic group with 5-year RS of 23.8% (95% CI 19.1% to 28.5%) for OSTT, 32.3% (95% CI 27.8% to 36.7%) for SCC, 43.5% (95% CI 36.9% to 50.1%) for ADC, 50.6% (95% CI 49.6% to 51.6%) for UC, 56.0% (95% CI 52.2% to 59.9%) for UTT, and 72.0% (95% CI 70.4% to 73.5%) for PIUC.

In addition, RS fell with increasing age at diagnosis. For example, RS for people aged 80+ years was 44.8% (95% CI 43.0% to 46.7%) whereas RS for people aged <60 years was 68.1% (95% CI 66.3% to 69.9%). Analysis of the topography demonstrated a most favorable relative 5-year RS of roughly 50% for the bladder (C67), ureter (C66), and kidney (C65), with the best survival of 57.2% (95% CI 56.4% to 58.1%) for the bladder. Other malignancies (C68: urethra, several overlapping areas, and unspecified localizations) showed poor survival with 33.8% (95% CI 29.5% to 38.1%).

**Figure 3:**
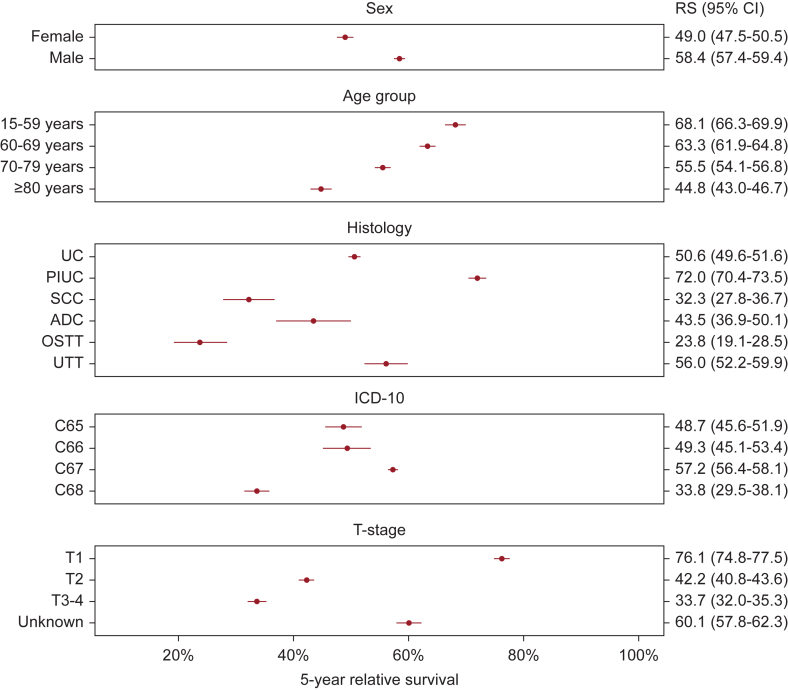
**Five-year relative survival of registered patients with malignant invasive urinary cancers within the calendar period 2018-2022 (*N* = 37 485), North Rhine-Westphalia, Germany.** ADC, adenocarcinoma; C65, renal pelvic; C66, ureter; C67, urinary bladder; C68, other and unspecified urinary organs; CI, confidence interval; OSTT, other specific tumor types; PIUC, papillary invasive urothelial carcinoma; RS, 5-year relative survival; SCC, squamous-cell carcinoma; UC, urothelial carcinoma; UTT, unspecified tumor types.

**Figure 4:**
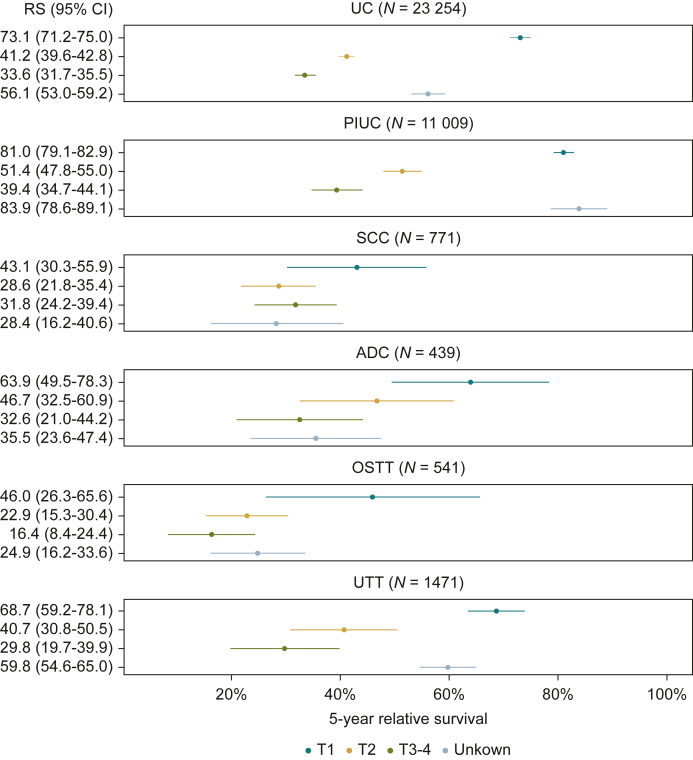
**Five-year relative survival of registered patients with malignant invasive urinary cancers by histology and tumor size within the calendar period from 2018 to 2022, North Rhine-Westphalia, Germany.** ADC, adenocarcinoma; CI, confidence interval; OSTT, other specific tumor types; PIUC, papillary invasive urothelial carcinoma; RS, 5-year relative survival; SCC, squamous-cell carcinoma; UC, urothelial carcinoma; UTT, unspecified tumor types.

Our data indicate that survival decreased from 76.1% (95% CI 74.8% to 77.5%) in T1 to 33.7% (95% CI 32.0% to 35.3%) in stages T3-4. Sex-specific analysis showed that RS was markedly worse for women at 49.0% (95% CI 47.5% to 50.5%) compared with men at 58.4% (95% CI 57.4% to 59.4%) (Figure 3). Furthermore, a decline in 5-year RS across T-stages was also observed for various histologic variants, particularly for UC, PIUC, ADC, and UTT (Figure 4). In contrast, for SCC, RS was 31.8% (95% CI 24.2% to 39.4%) for T3-4 and 28.6% (95% CI 21.8% to 35.4%) for T2. Notably, the best survival was observed for T1 PIUC with an RS of 81.0% (95% CI 79.1% to 82.9%), whereas patients with T3-4 and OSTT exhibited the poorest survival with an RS of 16.4% (95% CI 8.4% to 24.4%).

Absolute 5-year survival for the overall cohort, separated by age group and sex in the calendar period 2018-2022, is shown in Supplementary Table S1, available at https://doi.org/10.1016/j.esmorw.2025.100678. Worst absolute survival was observed in OSTT and SCC, while the best absolute survival was seen in PIUC.

## Discussion

Our results rely on a large population-based cohort of 73 751 patients with UC recorded in the NRW State Cancer Registry. The study aimed to investigate histologic tumor type variants of UCs and their incidence, survival, and mortality rates. To date, only limited analyses of histologic UC variant tumor types are available. The division is based primarily on morphologic features in hematoxylin–eosin-stained pathologic sections. UC subtypes always show urothelial differentiation in combination with specific morphologic phenotypes, while non-urothelial tumor types show independent features.^16^ Pure ADC shows no evidence of urothelial differentiation, while a concurrent UC *in situ* would render the tumor as an UC with glandular differentiation. In case of an SCC in the bladder, the 2022 WHO classification framework allows, in contrast to the situation in ADCs, a concurrent UC *in situ*. For example, squamous differentiation is observed in up to 18% of invasive tumors, while pure ADC accounts for ∼2% of all cases.^16^

Predominant histologies in our cohort were pure UC and PIUC, with the distribution of ADC, SCC, and OSTT being consistent with previous reports.^16^^,^^17^ In contrast to earlier studies, we analyzed ASRs over time. Our findings revealed minor fluctuations, with a slight decrease among men. Regarding tumor types, heterogeneous trends were observed, with a tendency toward a decline in UTT and an increase in OSTT. For ADC, the decrease was more pronounced among women than among men. Such fluctuations can only be demonstrated in large epidemiological studies such as ours. Conversely, the distribution of rare tumor types has remained unchanged in clinical practice. Furthermore, unlike data from the Surveillance, Epidemiology, and End Results (SEER) database, we did not observe a decline in UC incidence rates; instead, we found a stable trend with a marginal increase. Our results confirm the adverse impact of histologic variants on oncologic outcomes, particularly for SCC and OSTT, demonstrating a 5-year RS of 32.2% (SCC) and 23.8% (OSTT), in contrast to 72.0% for PIUC and 50.6% for UC. A study using data from the SEER registry examined the stage at presentation and survival of non-urothelial tumor types compared with UC. The group of UC-only patients was not further subdivided in the SEER analysis. Our results suggest that further classification of the UC histology group is relevant, because PIUC showed a better survival than either UC or non-urothelial tumor types.

In comparison, the results of the SEER registry also showed poor survival for SCC and neuroendocrine carcinomas; however, in contrast to our results, the worst overall survival was observed in SCC cases.^17^

Differences in perioperative therapy may contribute, to a certain extent, to the oncological outcome in patients with UC and histologic subtypes. In addition, the treatment landscape for metastatic UC has evolved in recent years with the approval of immune-checkpoint-inhibition therapies.^6^

Results from the SEER registry demonstrated poorer survival especially for SCC compared with UC alone.^17^ Our data confirm these results with the addition that OSTT is also associated with poorer survival. While the SEER study did not include sex-specific analyses, our results show clear sex differences, such as the relative frequency of unfavorable SCC (5.3% versus 1.1%) and favorable PIUC (18.7% versus 27.7%) or the poorer survival of women compared with men (5-year RS 49.0% versus 58.4%).

Furthermore, a decreasing ASR for PIUCs was observed for women and men, with an APC of –2.3 for women and –2.5 for men (2008-2022). It should be noted that the morphology code 8130/3 for PIUC is marked as obsolete in the current classification, which would explain the decreasing APC.^9^ The impact of tumor stage revealed a decreasing survival from 76.1% in T1 to 33.7% in stages T3-4. A decline in 5-year RS across T-stages was also observed for various histologic variants, particularly for UC, PIUC, ADC, and UTT.

In sex-specific analysis, women had a lower proportion of PIUC and a higher proportion of SCC and UTT compared with men. Furthermore, women showed a poorer absolute 5-year survival of 23.6% compared with 31.0% in men within the SCC group, which can be explained by the more aggressive histological tumor types but also more advanced disease. Our data show that the incidence rates of PIUC and UTT are decreasing, which could be partly due to the labeling of the morphology code for PIUC as obsolete and partly due to increasingly better reporting quality. Nevertheless, the data also show a very good RS and corresponding prognostic significance, so that recording PIUC should still be considered.

In the PURE-01 study investigating neoadjuvant therapy for muscle-invasive UC, dominant variant tumor types were not excluded and the majority of cases had SCC (37%).^8^ In a total of 19 patients with dominant variant histology, neoadjuvant administration of immune-checkpoint-inhibition therapy with pembrolizumab achieved downstaging in 42% and pathological complete response in 16%. The study results confirm the efficacy of neoadjuvant pembrolizumab in SCC patients who may be suitable for future neoadjuvant immunotherapy trials.^8^ Clearly, the poor clinical outcomes indicate the medical need in these patient populations.

The strength of this study is the use of population-based data from one of Europe’s largest cancer registries, providing data on incidence and survival of patients treated in Germany. Due to comprehensive mortality follow-up in the Cancer Registry of NRW, we were able to provide a detailed analysis using the RS approach, providing insights into the survival of variant histology with regard to different subgroups.

Some limitations of our analysis have to be acknowledged. The observed changes in incidence rates for certain histology groups may be influenced by changes in the reporting of unspecified histology, rather than true changes in incidence. Some of our survival estimates are based on small numbers and consequently survival may be imprecisely estimated, especially when further stratification factors were applied. T3 and T4 groups were also aggregated to ensure a more precise survival estimate. Our database does not contain more specific information on local or systemic therapy, so we could not consider this information. In particular, the lack of information on systemic therapy regimens is crucial for treatment comparisons. However, these clinical variables have been collected in our database since 2016, which will allow future analyses of treatment outcomes and comparisons.

In conclusion, our data demonstrate that UC and its variants exhibit diverse clinical outcomes. Survival declined with age, with SCC (32.3%) and OSTT (23.8%) showing particularly poor outcomes, whereas PIUC (72%) demonstrated more favorable results. Topographic analysis revealed better survival rates for bladder, ureter, and kidney malignancies, but poorer outcomes for urethra and unspecified localizations. The overall cohort and sex-specific analysis highlighted OSTT as having the worst survival outcome, with men exhibiting a rate of 17.2% and women 23.6% in the SCC group. Our data reveal a high medical need among patients with pure histologic subtypes, a group that has been systematically excluded from previous studies.
