# Supplementary material for: Histotype-specific incidence and survival of urothelial carcinoma—an analysis of the German North Rhine-Westphalia Cancer Registry
Source: ESMO Real World Data Digit Oncol. 2026 Jan 19;11:100678. doi: 10.1016/j.esmorw.2025.100678 (PMC13040895; doi:10.1016/j.esmorw.2025.100678)
Supplement: Supplementary Material [file mmc1.docx]

**Supplementary material:**

**Supplementary Figure 1 Annual age-standardized incidence rate of malignant invasive urinary cancers between 2008 and 2022, North Rhine-Westphalia, Germany ^*^**


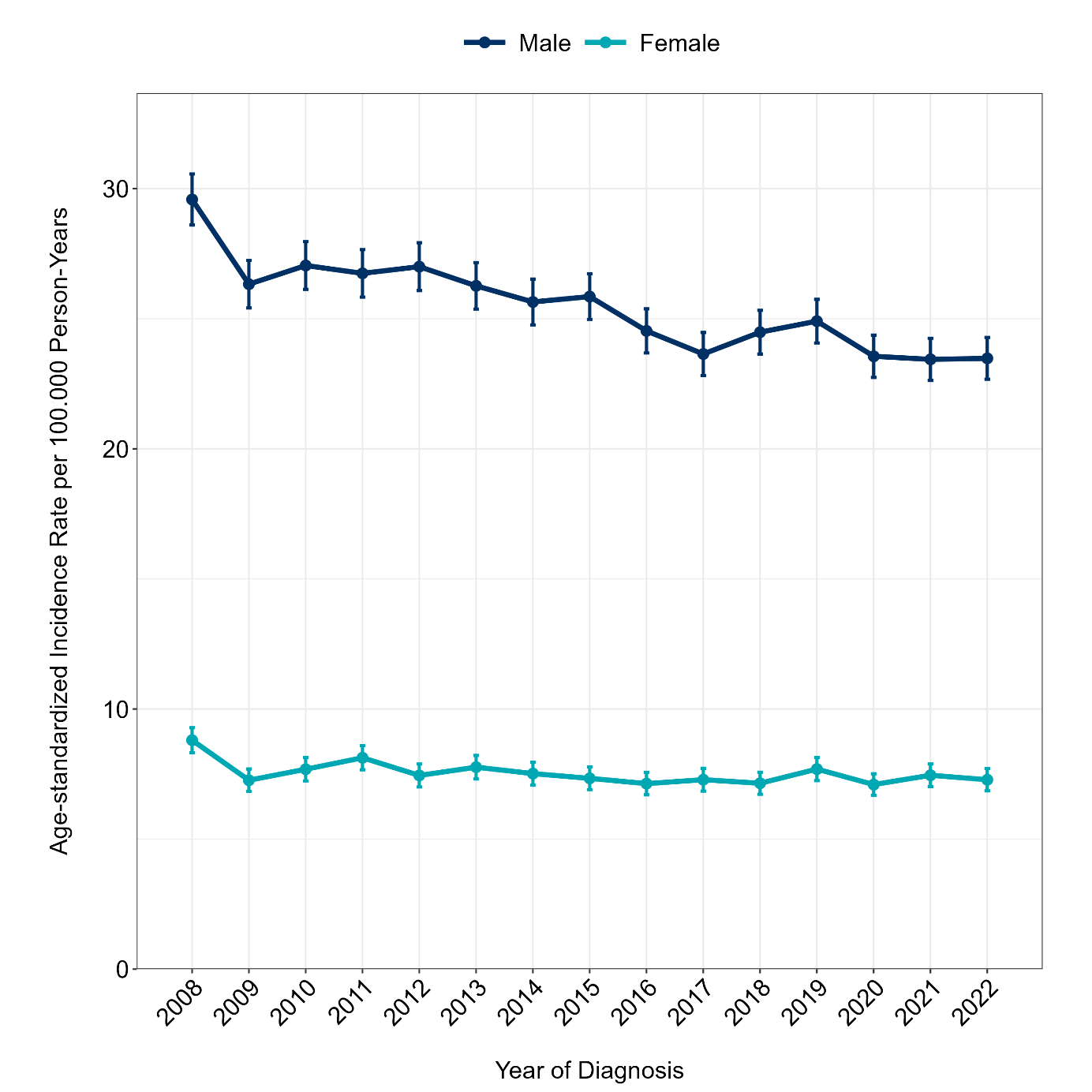


^*^ using the old European standard population

**Supplementary Table 1 5-year absolute survival of registered patients with malignant invasive urinary cancers (95% CI), calendar period 2018-2022, North Rhine-Westphalia, Germany**

|  | | **UC** | **PIUC** | **SCC** | **ADC** | **OSTT** | **UTT** |
| --- | --- | --- | --- | --- | --- | --- | --- |
| **Age group** | **Sex** | **AS (95% CI)** | **AS (95% CI)** | **AS (95% CI)** | **AS (95% CI)** | **AS (95% CI)** | **AS (95% CI)** |
| Overall | Male | 41.1 (40.2; 42.0) | 55.5 (54.1; 56.9) | 31.0 (24.8; 37.2) | 37.4 (30.5; 44.3) | 17.2 (12.7; 21.7) | 48.4 (44.6; 52.2) |
|  | Female | 36.1 (34.6; 37.6) | 58.1 (55.5; 60.7) | 23.6 (19.1; 28.1) | 33.8 (24.9; 42.7) | 24.6 (17.3; 31.9) | 39.1 (33.8; 44.4) |
| 15-59 years | Male | 65.1 (62.5; 67.7) | 79.9 (76.6; 83.2) | 49.6 (31.2; 68.0) | 58.4 (43.6; 73.2) | 39.4 (22.7; 56.1) | 74.0 (64.7; 83.3) |
|  | Female | 54.6 (50.2; 59.0) | 80.5 (74.3; 86.7) | 41.1 (28.0; 54.2) | 49.4 (31.1; 67.7) | 44.4 (22.1; 66.7) | 64.7 (49.9; 79.5) |
| 60-69 years | Male | 54.2 (52.2; 56.2) | 71.2 (68.7; 73.7) | 33.3 (19.7; 46.9) | 43.3 (29.3; 57.3) | 22.3 (11.8; 32.8) | 61.6 (53.7; 69.5) |
|  | Female | 50.4 (46.9; 53.9) | 71.8 (66.7; 76.9) | 37.9 (26.2; 49.6) | 41.9 (23.4; 60.4) | 28.9 (11.5; 46.3) | 52.5 (41.2; 63.8) |
| 70-79 years | Male | 40.9 (39.3; 42.5) | 56.1 (53.8; 58.4) | 26.9 (16.1; 37.7) | 29.1 (17.2; 41.0) | 16.2 (8.5; 23.9) | 50.4 (44.0; 56.8) |
|  | Female | 38.7 (36.0; 41.4) | 64.8 (60.3; 69.3) | 25.9 (16.8; 35.0) | 36.9 (16.4; 57.4) | 25.0 (12.1; 37.9) | 41.6 (31.6; 51.6) |
| ≥ 80 years | Male | 22.1 (20.6; 23.6) | 31.9 (29.5; 34.3) | 23.7 (13.2; 34.2) | 21.9 (9.7; 34.1) | 5.1 (0.2; 10.0) | 23.1 (17.2; 29.0) |
|  | Female | 20.5 (18.4; 22.6) | 35.4 (31.0; 39.8) | 7.2 (2.7; 11.7) | 10.9 (0.1; 21.7) | 12.3 (2.8; 21.8) | 19.4 (12.3; 26.5) |

UC = urothelial carcinoma, PIUC = papillary invasive urothelial carcinoma, SCC = squamous cell carcinoma, ADC = adenocarcinoma, OSTT = other specific tumor types, UTT= unspecified tumor types, AS = 5-year absolute survival
